# Impact of Idarucizumab and Andexanet Alfa on DOAC Plasma Concentration and ClotPro^®^ Clotting Time: An Ex Vivo Spiking Study in A Cohort of Trauma Patients

**DOI:** 10.3390/jcm10163476

**Published:** 2021-08-06

**Authors:** Daniel Oberladstätter, Christoph J. Schlimp, Johannes Zipperle, Marcin F. Osuchowski, Wolfgang Voelckel, Oliver Grottke, Herbert Schöchl

**Affiliations:** 1 AUVA Trauma Centre Salzburg, Department of Anaesthesiology and Intensive Care Medicine, Academic Teaching Hospital of the Paracelsus Medical University, 5020 Salzburg, Austria; wolfgang.voelckel@auva.at (W.V.); herbert.schoechl@auva.at (H.S.); 2 AUVA Trauma Research Centre, Ludwig Boltzmann Institute for Experimental and Clinical Traumatology, 1200 Vienna, Austria; christoph.schlimp@trauma.lbg.ac.at (C.J.S.); johannes.zipperle@trauma.lbg.ac.at (J.Z.); marcin.osuchowski@trauma.lbg.ac.at (M.F.O.); 3 AUVA Trauma Centre Linz, Department of Anaesthesiology and Intensive Care Medicine, 4010 Linz, Austria; 4 Department of Anaesthesiology, RWTH Aachen University Hospital, 52074 Aachen, Germany; ogrottke@ukaachen.de

**Keywords:** DOAC, reversal, Idarucizumab, Andexanet alfa, RVV-test, ECA-test

## Abstract

Specific antagonists have been developed for the reversal of direct oral anticoagulants (DOAC). We investigated the impact of these reversal agents on the plasma concentration and visco-elastic test results of dabigatran and factor Xa inhibitors. After baseline measurements of dabigatran, the plasma concentration, and the visco-elastic ClotPro^®^ ecarin clotting time (ECA-CT), we added the reversal agent Idarucizumab in vitro and these two analyses were repeated. Likewise, the baseline plasma concentration of apixaban, edoxaban, and rivaroxaban as well as ClotPro^®^ Russell’s viper venom test clotting time (RVV-CT) were measured and reanalyzed following Andexanet alfa spiking. We analyzed fifty blood samples from 37 patients and 10 healthy volunteers. Idarucizumab decreased the measured dabigatran plasma concentration from 323.9 ± 185.4 ng/mL to 5.9 ± 2.3 ng/mL and ECA-CT from 706.2 ± 344.6 s to 70.6 ± 20.2 s, (all, *p* < 0.001). Andexanet alfa decreased the apixaban concentration from 165.1 ± 65.5 ng/mL to 9.8 ± 8.1 ng/mL, edoxaban from 152.4 ± 79.0 ng/mL to 36.4 ± 19.2 ng/mL, and rivaroxaban from 153.2 ± 111.8 ng/mL to 18.1 ± 9.1 ng/mL (all *p* < 0.001). Andexanet alfa shortened the RVV-CT of patients with apixaban from 239.2 ± 71.7 s to 151.1 ± 30.2 s, edoxaban from 288.2 ± 65.0 s to 122.7 ± 37.1 s, and rivaroxaban from 225.9 ± 49.3 s to 103.7 ± 12.1 s (all *p* < 0.001). In vitro spiking of dabigatran-containing blood with Idarucizumab substantially reduced the plasma concentration and ecarin-test clotting time. Andexanet alfa lowered the concentration of the investigated factor Xa-inhibitors but did not normalize the RVV-CT. In healthy volunteers’ blood, Idarucizumab spiking had no impact on ECA-CT. Andexanet alfa spiking of non-anticoagulated blood prolonged RVV-CT (*p* = 0.001), potentially as a consequence of a competitive antagonism with human factor Xa.

## 1. Introduction

Direct oral anticoagulants (DOACs) are increasingly prescribed for stroke prevention in patients with non-valvular atrial fibrillation or as prophylaxis and treatment of thrombo-embolic events [1,2]. Phase III studies revealed a higher efficacy and an equivalent or lower rate of spontaneous bleeding events compared to vitamin K antagonists [3,4,5,6]. Similar findings have been reported for anticoagulated trauma patients on DOACs [7,8,9,10,11].

Recently, DOAC specific reversal agents have been approved, which considerably facilitate bleeding management in emergency scenarios. The humanized antibody Idarucizumab rapidly inactivates free, and clot-bound dabigatran with a high efficacy [12,13]. Andexanet alfa, a modified and catalytically inactive recombinant human factor Xa molecule, competitively inhibits the activity of apixaban and rivaroxaban [14], but has not been yet approved for edoxaban due to the lack of data.

Based on their predictable pharmacokinetics routine, monitoring of the anticoagulant potential of DOACs is deemed unnecessary [15]. In emergency situations such as trauma, acute operations, drug overdose or prior to thrombolysis in acute stroke, the measurement of DOAC plasma concentration is recommended [16,17,18]. However, analyses of DOAC plasma level are time-consuming and not available in all hospitals [19]. Thus, point-of-care visco-elastic devices, capable of DOAC monitoring have gained interest [20,21,22]. These tests can be run in citrated whole blood without a need for centrifugation and the first test results (e.g., clotting time) are available within minutes. A recent prospective observational study reported high correlations between clinically-relevant dabigatran and factor Xa-inhibitor plasma concentrations and the clotting time of these commercially available DOAC specific assays [22].

Studies on the impact of these DOAC specific antagonists on the plasma level before and after the treatment are still limited [12,13,14]. Moreover, no studies so far have investigated the effect of these reversal agents on DOAC specific visco-elastic test results. The aim of the current study was to examine in vitro the impact of Idarucizumab and Andexanet alfa on blood samples collected from trauma patients. Changes in plasma concentration and the clotting time using the DOAC-specific, visco-elastic assays ecarin-test for dabigatran and Russell’s viper venom-test for apixaban, edoxaban, and rivaroxaban, were investigated.

## 2. Materials and Methods

After obtaining approval from the AUVA Ethics Committee (approval number 09-2020), this prospective single-center study was conducted from July 2020 to February 2021 at the AUVA trauma centre Salzburg, Austria. All trauma patients >18 years with concomitant DOAC intake were eligible for inclusion. In order to investigate relevant DOAC concentrations for the spiking experiment, only blood samples from patients with an initial plasma DOAC level of > 50 ng/mL were included.

Measurement of the DOAC plasma concentration, using specific calibrators, is a standard diagnostic procedure at the AUVA trauma centre Salzburg for trauma patients on DOACs undergoing elective surgical procedures, and in all emergency scenarios such as an urgent surgery, trauma with risk of bleeding and patients with traumatic brain injury.

We drew blood samples for coagulation analyses upon hospital admission and/or subsequently during their hospital stay. A written informed consent was obtained from all participants.

### 2.1. Coagulation Analysis

#### 2.1.1. Measurement of DOAC Plasma Concentration

Blood samples were collected in 3 mL tubes containing buffered trisodium citrate 3.2% (S-Monovette; Sarstedt AG, Nürmbrecht, Germany), with a citrate:blood volume ratio of 1:9. After centrifugation (2500 G for 10 min), the DOAC concentration was measured in platelet-poor plasma on STA Compact Max 3 (Diagnostica Stago, Asnieres, France) device using specific chromogenic tests. For dabigatran, STA-ECA II (Diagnostica Stago, Asnieres, France) was used. For the measurement of the direct factor Xa-inhibitors apixaban, edoxaban, and rivaroxaban, the STA-Liquid Anti-Xa assay with dedicated calibrators (Diagnostica Stago, Asnieres, France) were applied. Moreover, the standard coagulation test prothrombin time (PT, reference limit 11–16.1 s; STA-NeoPTimal; Diagnostica STAGO, Asnieres, France) and activated partial thromboplastin time (aPTT, reference limit 24–35 s; Cephascreen; Diagnostica STAGO, Asnieres, France) were measured with the same device.

#### 2.1.2. DOAC Specific Visco-Elastic Analyses Assays

Citrated blood was used for simultaneous (within 1 h) visco-elastic testing on a ClotPro^®^ analyzer (Haemonetics, Braintree, MA, USA). ClotPro^®^ is a 6-channel device, where a cup with whole blood is set in alternating rotational motion and visco-elastic forces of blood clotting are detected via highly sensitive electronic sensors. Increasing clot stability results in an inhibition of the cup-rotation, which is traced as a curve over time. The DOAC specific reagents are kept in dried form in a sponge located in the tip of the pipette (Active tip^TM^).

Two different assays are used for DOAC measurements. An ecarin based test (ECA-test) is established for the detection of dabigatran. Ecarin, derived from the venom of Echis carinatus, activates prothrombin to meizothrombin that cleaves fibrinogen to fibrin. Dabigatran inactivates meizothrombin with high efficacy, thus prolonging the ECA-test clotting time (CT; refence limit 68–100 s). Clotting time is defined as the elapsed time from the addition of an activator until a clot amplitude of 2 mm is reached. The ECA-test is insensitive to factor Xa-inhibitors, as coagulation is initiated at the level of prothrombin. For the measurement of factor Xa-inhibitors, the Russell’s viper venom assay (RVV-test) was applied. The Russell’s viper venom is a potent factor X activator. Inhibition of factor Xa consequently prolongs the Russell’s viper venom-test clotting time (CT; reference limit 48–77 s).

A volume of 0.34 mL was removed from the primary blood sample for baseline visco-elastic testing on ClotPro^®^. The remaining blood volume (2.66 mL) was used for the spiking experiment. Blood samples containing dabigatran were spiked with Idarucizumab (Praxbind, Boehringer Ingelheim International GmbH, Ingelheim am Rhein, Germany). A volume of 25 µL/1.25 mg (eq. 84.6 mL/4.23 g at 4.5 L blood volume) was added to 1.33 mL of citrated blood, which corresponds to 5 g Idarucizumab, the recommended dose for reversal of dabigatran [12]. Blood samples containing one of the factor Xa-inhibitors were incubated with the specific antagonist Andexanet alfa (Ondexxya, Portola Netherlands B.V., Amsterdam, The Netherlands), and 25 µL/0.25 mg was added to 1.33 mL citrated blood (eq. 84.6 mL/846 mg at 4.5 L blood volume). The suggested dosage for Andexanet alfa in an adult is 400 mg (low dose) to 800 mg (high dose) as bolus-infusion followed by an infusion of 480 mg (low dose) to 960 mg (high dose) over 120 min [14]. After an incubation time of 5 min, another ClotPro^®^ analysis was performed.

The remaining blood samples were centrifuged (2500 G for 10 min) and the DOAC-concentration was measured, as previously described. All of the measurements were run within 1 h after blood sampling.

Additionally, we investigated the impact of Idarucizumab on the ECA-test clotting time and Andexanet alfa spiking and Russell’s viper venom-test clotting time on the prothrombin time and activated partial thromboplastin time in blood samples gathered from 10 healthy volunteers, without any history of DOAC intake. These tests were carried out as described above.

### 2.2. Statistical Analysis

Normality of the data was assessed using the D’Agostino and Pearson test. Continuous variables are expressed as mean and standard deviation (SD) or median and interquartile ranges (25th and 75th percentile). Categorical variables are reported as numbers and percentages (%). For analyzing differences of plasma concentration and clotting time before and after spiking with the specific antagonists Idarucizumab or Andexanet, the alfa paired *t*-test or Wilcoxon matched-pairs signed rank test was applied.

All of the statistical calculations were performed using GraphPad Prism (version 9.0.1., Graph-Pad Software, La Jolla, CA, USA). The level of significance was set at *p* < 0.05.

## 3. Results

A total of 50 blood samples (13 dabigatran, 11 apixaban, 13 edoxaban, and 13 rivaroxaban) were obtained from 37 patients. In seven patients more than one blood sample, drawn at different time-points, were included. Repeated measurements were due to a Dabigatran rebound or delayed plasma level reduction. The predominant indication for DOAC intake was atrial fibrillation. Five patients with operative therapy and 1 with TBI received red blood cells in the first 24 h (1–2 units). The median age of the predominantly female patients (59.5%) was 83.7 ± 10.0 years. Blood samples from 10 healthy volunteers were gathered and served as control. Demographic and clinical data of the patients are outlined in (Table 1).

**Figure 1:**
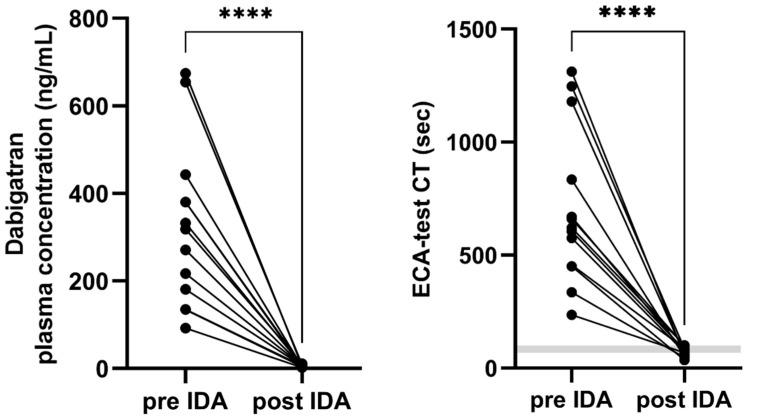
Dabigatran plasma concentration and ClotPro^®^ ECA-test CT before and after spiking with the specific dabigatran antagonist Idarucizumab. ECA-test: Ecarin based visco-elastic assay; CT: Clotting time; IDA: Idarucizumab; gray area represents normal limits of the specific test. **** *p* < 0.0001, paired *t*-test.

Spiking of dabigatran containing blood samples with Idarucizumab resulted in a significant decrease of both dabigatran plasma concentration and ECA-test clotting time. Dabigatran plasma levels declined from a mean of 324 ± 185 ng/mL to 6 ± 2 ng/mL. Only 1 measurement after Idarucizumab spiking revealed concentrations higher than 10 ng/mL. Similarly, Idarucizumab spiking of whole blood shortened the mean ECA-test clotting time from 706 ± 345 s to 71 ± 20 s. None of these measurements were outside of the upper reference limit (68–100 s) (Figure 1).

**Figure 2:**
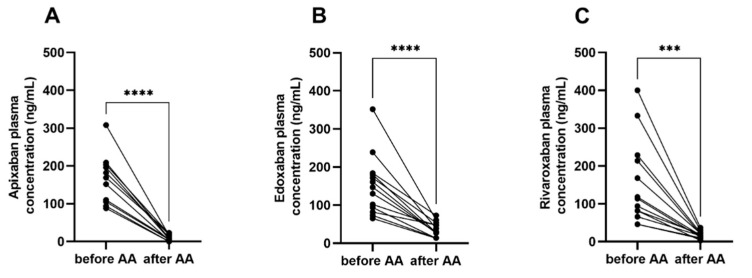
Factor Xa inhibitor. (**A**) Apixaban, (**B**) edoxaban, (**C**) rivaroxaban plasma concentration before and after spiking with the specific factor Xa-inhibitor Andexanet alfa. AA: Andexanet alfa; CT: Clotting time; RVV-test: Visco-elastic Russell’s viper venom assay. *** *p* < 0.001; **** *p* < 0.0001, paired *t*-test.

Spiking of Andexanet alfa to the citrated blood samples obtained from patients on factor Xa-inhibitors significantly decreased both plasma concentrations and Russell’s viper venom-test clotting time. For apixaban, the plasma concentration declined from 165 ± 66 ng/mL to 10 ± 8 ng/mL, for edoxaban from 152 ± 79 ng/mL to 36 ± 19 ng/mL, and for rivaroxaban from 153 ± 112 ng/mL to 18 ± 9 ng/mL (all *p* < 0.001). Andexanet alfa spiking resulted in a significant reduction of factor Xa-inhibitor concentration (94% for apixaban, 76% for edoxaban, and 88% for rivaroxaban). A total of three measurements after spiking with Andexanet alfa were above 50 ng/mL (Figure 2).

**Figure 3:**
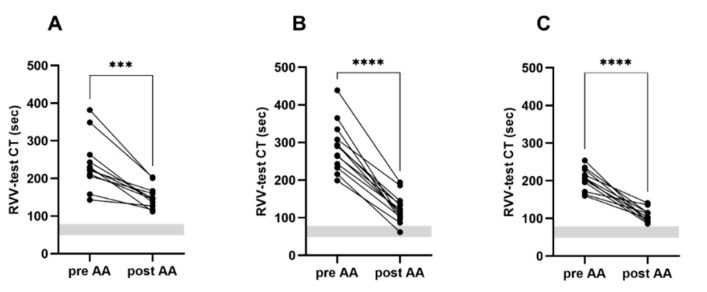
ClotPro^®^ RVV-test CT before and after spiking with the specific factor Xa-inhibitor Andexanet alfa. (**A**) Apixaban, (**B**) edoxaban, (**C**) rivaroxaban. AA: Andexanet alfa; CT: Clotting time; RVV-test: Russell’s viper venom assay; the gray area represents normal limits of the specific test. *** *p* < 0.001; **** *p* < 0.0001, paired *t*-test.

In contrast, Andexanet alfa decreased but did not normalize the Russell’s viper venom-clotting time for all three investigated factor Xa-inhibitors. Addition of Andexanet alfa shortened Russell’s viper venom-test clotting time in whole blood samples containing apixaban from 239 ± 72 s to 151 ± 30 s, in blood samples from patients on edoxaban from 288 ± 65 s to 123 ± 37 s, and in rivaroxaban containing whole blood from 226 ± 49 s to 104 ± 12 s. (all *p* < 0.001). All of the analyses except one were higher than the reference limits established by the manufacturer (48–77 s) (Figure 3).

**Figure 4:**
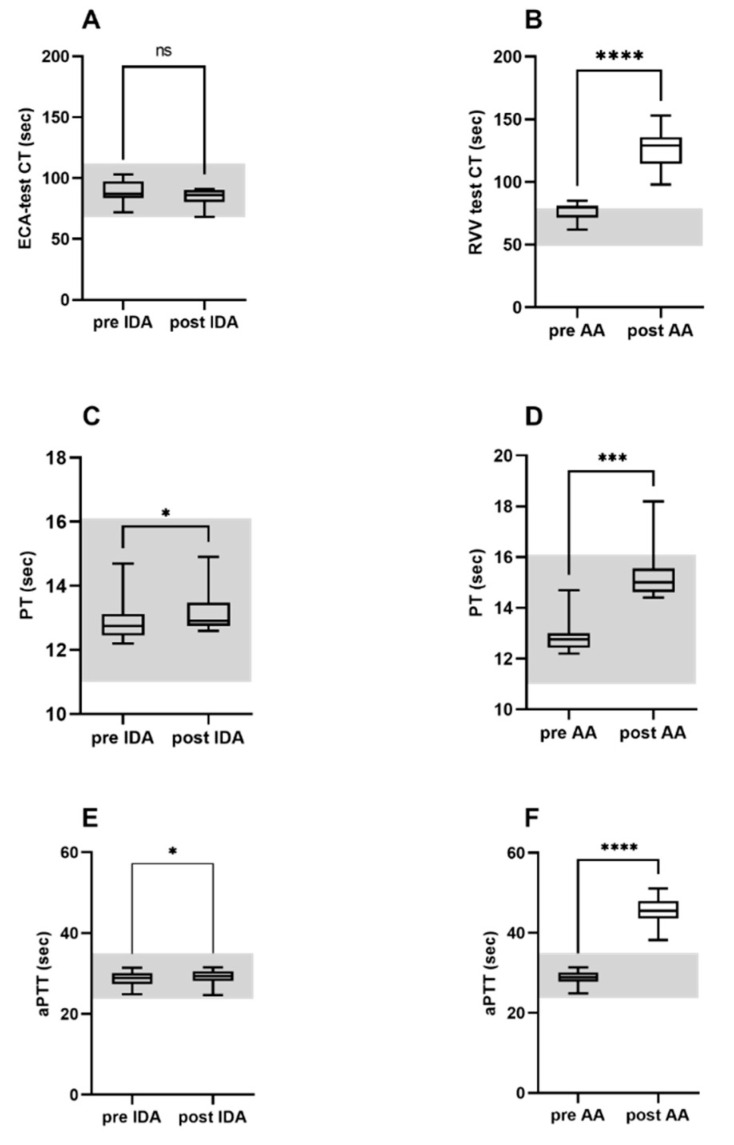
(**A**,**B**) ClotPro^®^ specific tests and (**C**–**F**) standard coagulation tests in healthy volunteers not on the antithrombotic therapy before and after spiking with Idarucizumab (**A**,**C**,**E**) and Andexanet alfa (**B**,**D**,**F**). AA: Andexanet alfa; IDA: Idarucizumab; RVV-test: Russell’s viper venom assay; ECA-test: Ecarin based assay; CT: Clotting time; PT: Prothrombin time; aPTT: Activated partial thromboplastin time; gray area represents normal limits of the specific test. * *p* < 0.05, *** *p* < 0.001, **** *p* < 0.0001, ns: Not significant; paired *t*-test.

Idarucizumab spiking of whole blood from healthy volunteers had a minor, though significant effect on the prothrombin time and activated the partial thromboplastin time, but had no significant effect on the ecarin clotting time. In contrast, an addition of Andexanet alfa to the volunteers’ blood significantly prolonged the prothrombin time, activated the partial thromboplastin time and Russell’s viper venom-test clotting time (Figure 4).

## 4. Discussion

We investigated the impact of the DOAC-specific reversal agents Idarucizumab and Andexanet alfa on both the plasma concentration and visco-elastic parameter clotting time. The current study revealed that spiking of the whole blood samples containing dabigatran with its specific antagonist Idarucizumab lowered the dabigatran plasma concentration with a high efficacy. Likewise, the ClotPro^®^ parameter ECA-test clotting time declined to the normal reference range. The addition of Andexanet alfa to blood samples incorporating factor Xa-inhibitors decreased the factor Xa-inhibitor plasma concentration to levels < 50 ng/mL in the majority of the analysed blood samples. In contrast, Andexanet alfa spiking of the whole blood reduced but did not normalize Russell’s viper venom-test clotting time. All of these measurements (except one) were outside the established reference limits. Spiking of non-anticoagulated whole blood with Idarucizumab had no impact on the ECA-test clotting time and only a minor influence on the standard coagulation test, but significantly prolonged Russell’s viper venom-test clotting time, aPTT. Additionally, the reduced PTI occurred when the whole blood was incubated with Andexanet alfa.

The availability of specific DOAC antagonists significantly facilitates the patient’s care in emergency scenarios where an immediate antithrombotic reversal is indicated. Data from the REVERS-AD study revealed that the treatment of bleeding patients on dabigatran with Idarucizumab resulted in a normal haemostasis in 93.4% of the patients [12]. Comparable results were reported for Andexanet alfa for emergency reversal of life-threatening bleeding events in patients with a prior intake of apixaban and rivaroxaban, but not yet for edoxaban [14].

Measurement of the DOAC plasma concentration prior to the invasive procedures remains a matter of debate. For example, the fifth edition of the European guidelines on the management of major bleeding and coagulopathy following trauma recommend measuring DOAC plasma level in injured patients [16]. In patients with acute ischemic stroke, a DOAC plasma level < 50 ng/mL was established as a cutoff for acute thrombolysis [23]. However, until now, only limited data are available regarding the usefulness of these analyses to improve the patient outcomes [24]. Moreover, despite the ever increasing number of patients on DOACs, many hospitals fail to offer these measurements [18].

A promising alternative are DOAC-specific whole blood tests, which can be run on visco-elastic analysers [20,21]. Our group recently showed that visco-elastic testing with the DOAC specific assays, ecarin-test for dabigatran, and Russell’s viper venom-test for factor Xa inhibitors, allow a quick and reliable estimation of clinically-relevant DOAC plasma concentrations [22].

According to the current guidelines, the administration of reversal agents in bleeding patients on DOACs should be considered whenever a plasma concentration exceeds 50 ng/mL [17]. However, the laboratory assessment of the efficacy of DOAC reversal remains controversial. Until now, only a few real-life studies reported the DOAC plasma concentration before and after reversal with specific antagonists [12,13,14]. Data from the REVERS-AD study revealed that immediately after Idarucizumab administration, the dabigatran plasma concentration returned to a low level and remained low for the next 24 h [12]. However, some case reports provided evidence that a single dose of 5 g Idarucizumab might not be sufficient in patients with a very high initial dabigatran plasma concentration. These subjects are at risk for a redistribution of dabigatran from the interstitial space into the intravascular compartment with a significant Idarucizumab increase at a later time-point [25,26]. For patients with a very high initial dabigatran concentration or renal insufficiency, repeated measurements of dabigatran are mandatory. In this setting, the ClotPro^®^ ECA-test provides a rapid and reliable tool for a frequent monitoring of Idarucizumab plasma concentrations. The current study revealed that Idarucizumab spiking returned the dabigatran plasma concentration to values <10 ng/mL and all ECA-test clotting times were within their reference limits, despite the fact that in some patients the dabigatran level prior to Idarucizumab spiking was extremely high.

In contrast, the quantification of a sufficient factor Xa-inhibitor reversal by Andexanet alfa is more challenging. Data from the ANNEXA-A, ANNEXA-R, and ANNEXA 4 studies revealed that reversal of rivaroxaban and apixaban with Andexanet alfa significantly reduced the activity of both factor Xa-inhibitors [14,27]. However, the validated chromogenic assay of factor Xa enzymatic activity used in those studies is not widely available. Importantly, the European Medicines Agency (EMA) approval summary for Andexanet alfa pointed out that commercial anti-factor Xa-activity assays should not be used for measuring the anti-factor Xa activity following the administration of Andexanet alfa. These assays provide an inaccurately elevated anti-factor Xa concentration, which may considerably underestimate the reversal effect of Andexanet alfa [28]. In contrast to the EMA’s statement, our study revealed that Andexanet alfa spiking resulted in a significant reduction of factor Xa-inhibitor concentration between 76% and 94% from baseline values when the STG-ECA test was applied.

Despite the fact that our spiking experiment of the whole blood samples with Andexanet alfa resulted in a significant decline of factor Xa-inhibitor concentration; almost all of the measurements using the RRV-test clotting time were outside of the reference limits established by the ClotPro^®^ manufacturer. Notably, Andexanet alfa spiking of healthy blood samples also significantly increased Russell’s viper venom-test clotting time and aPTT and reduced PTI. A potential explanation for this unexpected finding is that Andexanet alfa, which is chemically very similar to endogenous factor Xa, exerts a competitive antagonism resulting in a partial inhibition of the coagulation process, thus prolonging the initiation of the coagulation process in vitro.

It is important to point out that DOAC specific ClotPro^®^ assays were initially developed for a detection of clinically-relevant DOAC concentrations in whole blood but not as a monitoring tool for DOAC reversal therapy. However, the ECA-test clotting time perfectly corresponds with the dabigatran plasma concentration and can certainly be used to monitor the inhibition of dabigatran by Idarucizumab. In contrast, the association between the factor Xa-inhibitor plasma concentration and Russell’s viper venom-test clotting time is less distinct. A control of the therapeutic effect of Andexanet alfa, using established reference limits for Russell’s viper venom-test clotting time is not possible. Higher clotting time ranges, which we observed after Andexanet alfa spiking in the healthy volunteers’ blood, may constitute a target range for the factor Xa-inhibitor reversal by Andexanet alfa. However, this finding has to be confirmed in clinical studies.

## 5. Limitations

The current study relies solely on an in vitro experiment. Thus, it remains to be elucidated whether in vivo DOAC reversal therapy by the specific antagonists investigated here, provides similar results. The mode of action differs between Idarucizumab and Andexanet alfa. Idarucizumab rapidly inhibits free and a clot-bound dabigatran, whereas Andexanet alfa acts as a competitive decoy for factor Xa-inhibitors. Therefore, it is highly suggestive that an Idarucizumab administration will deliver comparable effects in vivo. Regarding the factor Xa-inhibitor reversal by Andexanet alfa, the results are less clear. However, it may be speculated that the Russell’s viper venom-test clotting time will decline following Andexanet alfa administration, but reference limits have to be established.

## 6. Conclusions

In vitro monitoring of the reversal effect of DOAC-specific antagonists was feasible with both measurement of the DOAC plasma concentration using the chromogenic test and with the DOAC-specific assays. Idarucizumab strongly inhibited the antithrombotic property of dabigatran resulting in almost unmeasurable dabigatran plasma concentrations and an ECA-test clotting time within normal limits. Factor Xa-inhibitor reversal by Andexanet alfa decreased the plasma concentration to <50 ng/dL in the majority of analyzed samples, but did not return Russell’s viper venom-test clotting time to normal limits. This may be related to a potential competitive mechanism between Andexanet alfa and the endogenous factor Xa resulting in an inhibition of the initiation phase of the coagulation process. Whether the in vitro control of antagonisation of DOACs may relate to the prediction of clinical reversal of bleeding should be subject to further studies.

## Figures and Tables

**Table 1:** Characteristics of patients on DOACs (*n* = 37).

Characteristics of Patients on DOACs	
Age, Years	83.7 ± 10.0
Female	22 (59.5)
Injury severity score	9 ± 3
Patterns of Injury	
Hip fractures	12 (32.4)
Limb trauma	6 (16.2)
Periprosthetic fractures	3 (8.1)
Serial rip fractures	2 (5.4)
Spine and pelvic fractures	4 (10.8)
Soft tissue trauma	5 (13.5)
Others	5 (13.5)
Indications for DOAC Therapy	
Atrial fibrillation	30 (81.0)
Deep vein thrombosis	3 (8.1)
Pulmonary embolism	2 (5.4)
Others	2 (5.4)
Distribution of DOACsS (Patients)	2 (5.4)
Dabigatran	7 (18.9)
Apixaban	9 (24.3)
Edoxaban	9 (24.3)
Rivaroxaban	12 (32.5)

Values are given as mean ± standard deviation or number (percentage).

## Data Availability

The data presented in this study are available on request from the corresponding author.
